# Influence of Electrostatic Precipitator Ash “Zolest-Bet” and Silica Fume on Sulfate Resistance of Portland Cement

**DOI:** 10.3390/ma13214917

**Published:** 2020-11-01

**Authors:** Yury Barabanshchikov, Kseniia Usanova, Stanislav Akimov, Aleksandr Uhanov, Andrej Kalachev

**Affiliations:** 1 Institute of Civil Engineering, Peter the Great St. Petersburg Polytechnic University, 195251 St. Petersburg, Russia; ugb@mail.ru (Y.B.); akimov_sv@spbstu.ru (S.A.); 2 Provcement-Vector JSC, 195251 St. Petersburg, Russia; uav@profcement.ru (A.U.); ak@profcement.ru (A.K.)

**Keywords:** concrete, cement, fly ash, strength, thermal expansion, shrinkage, concrete density, sulfate resistance, silica fume

## Abstract

The influence of the electrostatic precipitator ash “Zolest-bet” and silica fume on the sulfate resistance of Portland cement was studied. The evaluation criteria were the expansion, strength, density, and appearance of the samples, hardened in a 5% Na_2_SO_4_ solution starting from 3 days of age for 192 days at a temperature of 20 °C and 148 days at 40 °C. The mixture with the silica fume additive had the minimum expansion under the Na_2_SO_4_ action, and the mixture with the fly ash “Zolest-bet” additive had the greatest expansion. Zolest-bet ash in pure water shrank the mixture by 0.19 mm/m by the end of the hardening period, and it gave a linear expansion of 0.23 mm/m in a Na_2_SO_4_ solution after hardening. The mixture can be considered sulfate resistant at a given value of linear expansion. Despite the greatest expansion, the compressive strength of the samples with the Zolest-bet additive was found to be the highest at hardening in both environments. The flexural strength was found to be the highest after being in Na_2_SO_4_ solution. The sulfate resistance of the mixture with silica fume was higher than that of the mixture based on sulfate-resistant cement. This mixture did not have expansion in comparison with the initial length, instead it shrank, while the expansion of sulfate-resistant cement was 0.006% over the control period. The compressive strength of the mixture with the silica fume additive was slightly inferior to the strength of the mixture with Zolest-bet ash.

## 1. Introduction

The article contains research on sulfate resistance as a critical aspect of durability. Several studies have confirmed that durability is critical for building materials [1,2,3]. Fly ash is used as a partial replacement for Portland cement to improve the strength and sulfate resistance of concrete [4,5,6], as well as to obtain artificial aggregates [7,8]. There is evidence of a significant decrease in the diffusion rate in places where fly ash is present, as well as stabilization of calcium aluminate hydrates, which leads to an increase in resistance to sulfate attack. The results are consistent for cements with a C_3_A content of 9 to 12% [9].

Fly ash is usually replaced by 15–35% of cement by weight. This range is acceptable for replacement without a significant negative impact on concrete performance. Fly ash is divided into low-calcium fly ash with a CaO content of no more than 18% (class F) and high-calcium fly ash with a CaO content of more than 18% (class C) according to ASTM C618 [10].

In scientific publications, such as [11,12,13] and others, other additives are known that are used in lightweight concrete and affect their performance. The properties of structures made on the basis of concrete with similar additives were studied in [13,14].

Low-calcium fly ash permanently improved the sulfate resistance of mortar based on Portland cement with high C_3_A content, and generally met the criteria for high sulfate resistance when containing 2 to 25% fly ash as cement replacement by mass. Fly ash with a high calcium content (i.e., CaO > 20%) had very different properties. The authors showed that fly ash replacement of 20% to 40% resulted in increased expansion when compared to high C_3_A cement used alone. Mortars with different levels of high-calcium fly ash content can be produced in accordance with the criteria for high sulfate resistance. This can be achieved by using low-C_3_A Portland cements or ternary cement mixtures containing low levels of silica fume. [15]. However, fly ash with a similar chemical composition could have different physical characteristics [16], depending on the type and source of fuel, boiler system, and cooling system. In [17], 15 types of class C fly ash and 11 types of class F fly ash were investigated. Fly ash of class C contained from 19.5 to 32.5% CaO with an average diameter D_50_ of 1.3 to 3.1 microns, and fly ash of class F contained from 2.1 to 17.1% CaO with D_50_ = 1.6–2.4 microns. In [18], a high-strength self-compacting concrete mix with high-calcium (class C) fly ash was designed. The compressive strength of the concrete was 100 MPa. It was found that with an equivalent w/b ratio, high-strength self-compacting concrete develops considerably higher compressive strength (more than 15 MPa) compared to that of conventionally vibrated high-strength concrete.

Estonian oil shale has a special place among the sources of fuel ash. It is characterized by a high content of carbonate minerals. Therefore, shale ash contains a lot of free lime. The properties of shale ash depend on the chemical and mineral composition, which in turn depend on the combustion temperature. A temperature of 800 °C is for low-temperature circulating fluidized bed firing technology and 1400 °C is for high-temperature powdered fuel combustion. The pulverized fuel ash from electrostatic precipitators of high-temperature boilers is used as an additional binder in cement materials [19]. This type includes the fly ash “Zolest-bet”. This ash of burnt oil shale is supplied by Narva to the entire European part of Russia and beyond the Urals.

The research in [20] was done to compare two types of shale ashes from the Narva power plant. The first type was cyclone ash and the second one was electrostatic precipitator ash. Both types had almost the same chemical composition. The cyclone ash significantly reduced the mechanical strength of the material and the electrostatic precipitator ash showed little change in the compressive strength and flexural strength. This may be due to its high dispersion. The electrostatic precipitator ash reduces the resistance of the material to solutions of sulfate salts more than cyclonic ash. However, at the same time, both ashes do not affect the frost resistance of the material.

The Jordan Natural Resources Authority considers oil shale as one of the most important promising energy sources, and shale ash as an alternative to other types of pozzolana. The authors compared the compressive strength of cement mortar cubes and mortar cubes with cement and fly ash. Replacement levels of fly ash were 10, 20, and 30% by weight. The replacement of cement by oil shale ash reduced the compressive strength of the cubes by 7.4, 11.7, and 23%, respectively. The setting time of cement paste increased by 20, 30, and 50 min, and the soundness also increased by 0, 0.5, and 1.5 mm due 10, 20, and 30% replacement of cement by oil shale ash, respectively. The experimental results confirmed that use of oil shale ash up to 20%, as there was no significant effect on the cement properties [21].

The research in [22] showed that an increase in the dispersion of shale ash significantly improves the rheology of the mixture and the rate of strength growth.

The study in [23] showed that free lime did not affect normal consistency and water demand, and free lime content up to 4.5% had little chemical effect on mixtures with cement and fly ash, i.e., faster setting, higher early age compressive strength, and higher autoclave expansion. However, the autoclave expansion values were still within the standard range. In terms of durability, high free lime fly ash mixtures resulted in higher expansion due to alkaline aggregate reactions.

In addition to the chemical composition, morphology, and crystallinity, particle size has a great influence on the change in the hydration, which, in turn, leads to a change in the mechanical properties and durability of fly ash concrete [24,25]. The paper [26] notes that the processing type, rather than the chemical composition, has a stronger effect on the compressive strength. The research in [27] shows that curing conditions are the most important factor affecting the durability of fly ash concrete. The effect of curing on cementitious concrete is of much lesser importance.

Investigating the action of two types of superplasticizers, the authors of [28] established that both types of superplasticizers were significantly less effective with geopolymers, compared with Portland cement paste. Polycarboxylate-based superplasticizer was more effective with class C than with class F fly ash. The naphthalene-based superplasticizer did not show much difference when added to the two types of fly ash.

There is conflicting information in the literature regarding the characteristics of fly ash, in particular concerning shrinkage, high-temperature curing, water requirements, toxicity [29], etc. Due to the high variability of characteristics observed for mortars and concretes containing fly ash with high calcium content, there is a need to test individual specific combinations of materials.

The objective of the work is the determination of the impact of electrostatic precipitator ash “Zolest-bet” and micro silica on the sulfate resistance of Portland cement in comparison with the sulfate resistance of a solution based on sulfate-resistant cement.

## 2. Materials and Methods

### 2.1. Testing Laboratory

Concrete with fly ash was tested in the Polytech-SKiM-Test laboratory of Peter the Great St. Petersburg Polytechnic University (Saint Petersburg, Russia).

### 2.2. Concrete Materials

For the production of concrete, the following materials were used:

1. Two types of Portland cement produced by OJSC Mordovcement (Mordovia, Russia).
  ― Portland cement CEM I 42.5 N.
  ― Sulfate-resistant Portland cement CEM I 32.5 SR5.
  The mineralogical composition of the cement is presented in Table 1.
2. Natural sand from the Ostrovskoye deposit (Leningrad region, Russia). The fineness modulus is from 2 to 2.5.
3. Silica fume MKU-85 produced by Yurga division of Kuznetskie Ferrosplavy (Yurga, Russia).
4. The oil shale fly ash additive “Zolest-bet” is produced by Enefit Energiatootmine AS (Narva, Estonia) in two oil shale-fired thermal power plants, Eesti Power Plant (Eesti Elektrijaam) and Balti Power Plant (Balti Elektrijaam). This fly ash is a mineral residue from the combustion of oil shale at temperatures of about 1300–1400 °C. The average grain size is 12–25 microns. The bulk density is 1100 ± 50 kg/m^3^. The specific surface area is 300–350 m^2^/kg. The true density is 2850 ± 50 kg/m^3^. The ash consists of C_2_S, CA, CaO_free_, and CaSO_4_ predominantly in the form of glass with latent bonding properties.

The chemical composition of the electrostatic precipitator ash “Zolest-bet” is presented in Table 2.

This fly ash is classified as class C (high CaO content) according to ASTM C618.

### 2.3. Concrete Mix Proportion

The mortar properties may vary slightly depending on the sequence of the addition of ingredients [30]. In this study, the standard mixing method was chosen. Concrete mixtures were produced using a standard concrete mixer for 90 s. The concrete mixer E093 manufactured by Matest (Arcore, Italy) was used in the test. Concrete mixtures are presented in Table 3.

The prepared mixtures were used in the flow test with an initial diameter of 100 mm after 30 shakes (see Table 3).

**Figure 1:**
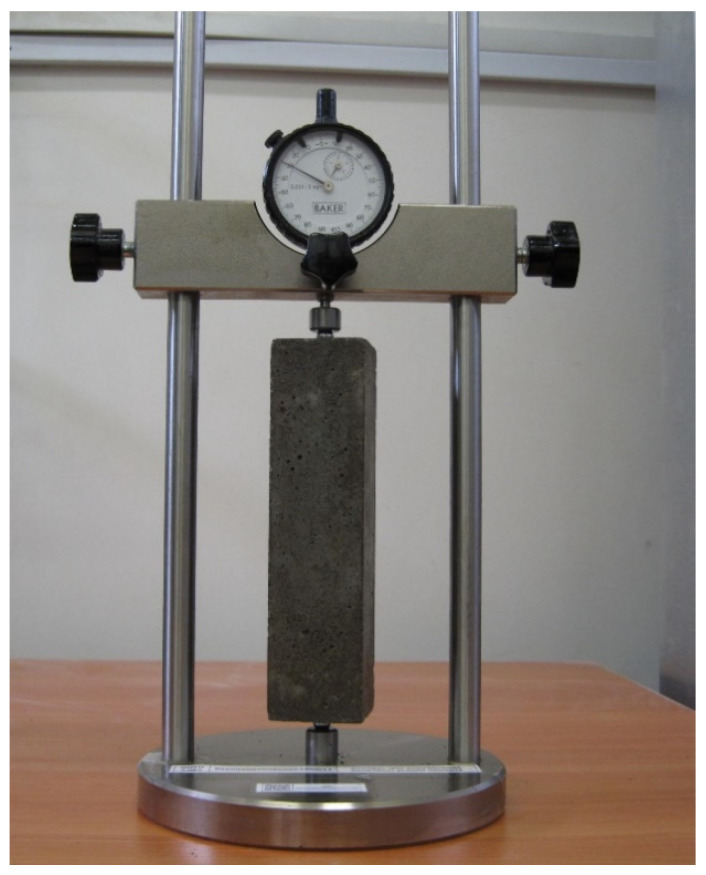
Device for measuring sample deformation.

From each mix, six beam samples with a size of 40 × 40 × 160 mm were made. Samples were made in special molds, making it possible to lay metal inserts at the ends of the samples for measuring shrinkage and thermal expansion. The samples were removed 1 day after production. The samples were stored in a normal hardening chamber for the first 3 days at a temperature of 20 ± 2 °C and a relative humidity of 95 ± 5%. After that, the initial length of the sample was measured using a device with a Mercer clock gauge with a scale division of 1 μm (Figure 1).

Then, three samples of each mixture were stored in distilled water, and the other three samples were stored in a 5% Na_2_SO_4_ solution. Distilled water and sodium sulfate solution were periodically replaced. Undistilled water was also available for the test. However, it would have reduced the leaching rate. In addition, the composition of undistilled water may have differed from case to case. Therefore, distilled water was chosen for experiments as a reference for comparison.

During hardening, the increment in the length of the samples was measured at certain time intervals according to Russian State Standard GOST R 56687-2015 “Protection of concrete and reinforced-concrete constructions from corrosion. Test method of sulfate resistance of concrete”. After holding for 6 months, the compression strength and flexural strength of the samples were determined according to Russian State Standard GOST “Cements. Methods of bending and compression strength determination”.

## 3. Results and Discussion

### 3.1. Shrinkage and Expansion Test Results

**Figure 2:**
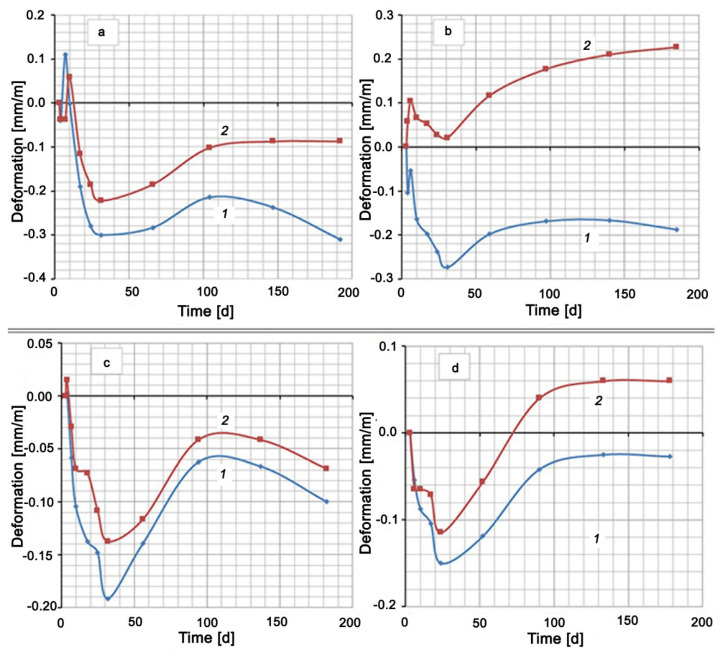
Shrinkage and expansion of compositions in time: (**a**) Control Mix; (**b**) mix with Zolest-bet additive (Mix No. 1); (**c**) mix with silica fume additive (Mix No. 2); (**d**) sulfate-resistant Portland cement (Mix No. 3); 1—at water hardening; 2—at 5% Na_2_SO_4_ solution hardening.

The hardening of all cement mixtures in water was accompanied by shrinkage (see Figure 2). An exception was the Control Mix, which had a short-term expansion to 0.11 mm/m on the seventh day of hardening. The hardening of the mixtures based on Portland cement CEM I 42.5 N (Control Mix, Mix No. 1, Mix No. 2) in 5% Na_2_SO_4_ solution showed at first a slight expansion (no more than 0.1 mm/m), which then changed to shrinkage. After about 25–30 days from the moment of mixing, the expansion of all samples, which hardened both in water and in a Na_2_SO_4_ solution, began. This expansion of mixtures in water reached its maximum value by 120–140 days, after which shrinkage began again. A similar maximum on the deformation curve among the compositions in sodium sulfate solution took place only for the mixture with the addition of silica fume (Mix No. 2). Other mixtures expanded until the end of the test. The compositions of Control Mix and Mix No. 2 had curves No. 2, which remained in the negative region (Figure 2a,c). This means that the samples, both in water and in Na_2_SO_4_ solution, showed shrinkage deformation at the end of the test period (Control Mix and Mix No. 2). The other two compositions, Mix No. 1 and Mix No. 3, underwent shrinkage in water and expansion in the Na_2_SO_4_ solution.

A small step on the deformation curves, and even expansion in the case of Control Mix and Mix No. 1, were observed in the initial section of shrinkage development. This circumstance and the subsequent wavelike nature of deformation indicated competition between contraction shrinkage and expansion under the action of the formed compounds and ordinary swelling in water.

The extent of the effect of Na_2_SO_4_ on expansion in comparison with pure water can be seen in the area between curves 1 and 2, the values of which for 180 days are given in Table 4.

Table 4 shows that the mixture with silica fume additive (Mix No. 2) has the smallest discrepancy between deformation in Na_2_SO_4_ solution and deformation in water, and, therefore, this mixture has the highest sulfate resistance. The mixture with the Zolest-bet additive (Mix No. 1) has the largest discrepancy between curves 1 and 2. The mixture based on sulfate-resistant cement (Mix No. 3) has, as expected, less expansion than the Control Mix based on Portland cement, but this expansion is 2.3 times greater than the expansion of the mixture with silica fume additive (Mix No. 2).

**Figure 3:**
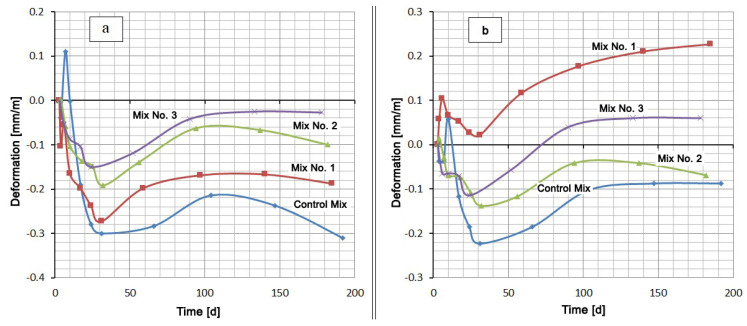
Shrinkage and expansion of compositions in time: (**a**) at water hardening; (**b**) at 5% Na_2_SO_4_ solution hardening.

A comparison of the mixtures by the deformation value in the same medium gives a slightly different picture (Figure 3). Typically, cementitious compositions swell when hardened in water. However, in this case, the samples were placed in water 3 days after production, when a sufficiently rigid skeleton of the material was formed. In this case, the penetration of moisture inside is difficult. At this age, the most intense contraction shrinkage of the material, which overlaps the swelling, occurs. After about 30 days of hardening, the shrinkage rate decreases and the expansion process begins to prevail. Despite this, the deformation values of all mixtures remain in the negative region (shrinkage region) (see Figure 3a). Under these conditions, sulfate-resistant Portland cement CEM I 32.5 N SR5 has the lowest shrinkage, and the Control Mix based on Portland cement CEM I 42.5 N has the highest shrinkage. At the same time, replacing 20% of the cement in the Control Mix by Zolest-bet additive (Mix No. 1) reduced the final shrinkage by almost 40%. A similar replacement of cement by silica fume reduces shrinkage by more than three times.

The mix with Zolest-bet additive (Mix No. 1) in 5% Na_2_SO_4_ solution showed the highest expansion value (0.227 mm/m) at 180 days. Throughout this period, the deformation values of the mix with Zolest-bet additive were positive. The mix based on sulfate-resistant Portland cement remained in the shrinkage area until 70 days, and after that, it began to exceed the initial sample size. The final expansion of this mix was 0.06 mm/m. The best results were obtained for the mix with silica fume additive (Mix No. 2), the expansion of which did not go beyond the negative region. Additionally, in the area of shrinkage, the deformation of the Control Mix remained, but its shrinkage turned out to be higher than that of the mix with silica fume additive.

**Figure 4:**
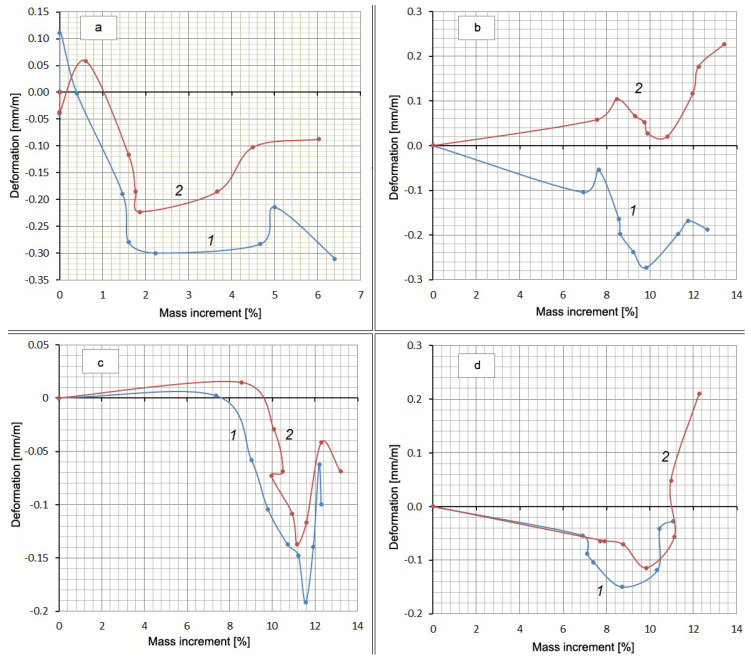
Deformation of compositions depending on the amount of absorbed substance: (**a**) Control Mix; (**b**) mix with Zolest-bet additive (Mix No. 1); (**c**) mix with silica fume additive (Mix No. 2); (**d**) sulfate-resistant Portland cement (Mix No. 3); 1—at water hardening; 2—at 5% Na_2_SO_4_ solution hardening.

Along with measuring the deformation, the mass increment of the samples was determined by weighing them on an electronic balance with a scale division of 0.01 g in the same periods. It was obvious that the increase in the mass of the samples occurred due to the absorption of either water or a Na_2_SO_4_ solution. The reverse process (weight reduction) could have occurred because of Ca(OH)_2_ leaching. A definite relationship between the expansion and the amount of absorbed water or solution must have existed. However, the experiments did not show a stable correlation between these values, but there was something in common in the form of all curves in the form of a “pit” or a loop in the middle of the graph (Figure 4).

**Figure 5:**
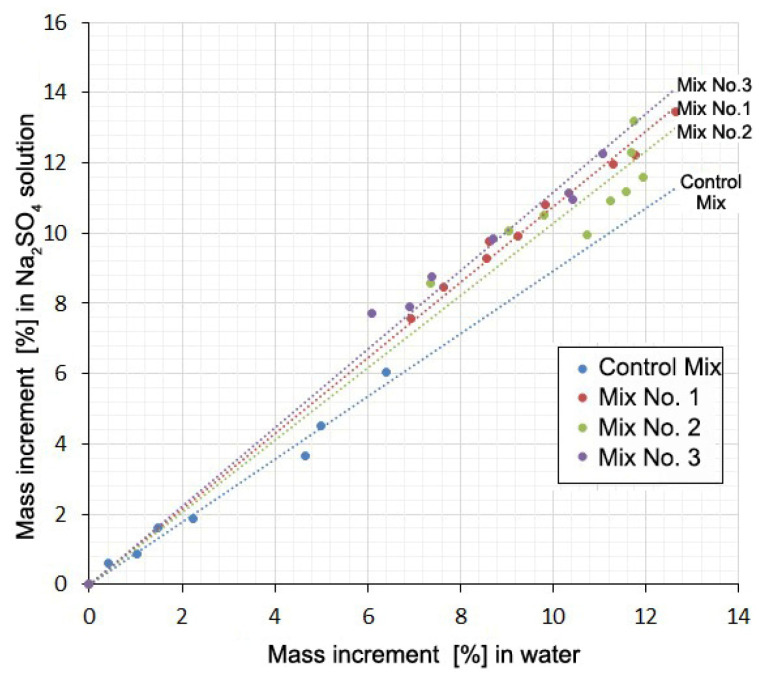
Mass increment of samples in Na_2_SO_4_ solution as a function of the mass increment in water at the same times.

The falling part of the loop indicates that shrinkage occurs despite the increase in the mass of the samples. Additionally, there is some similarity between curves 1 and 2 for each mixture. This suggests a possible correlation between the deformation values or mass increments for the same mixtures at the same time points. This correlation in the form of direct proportionality is obtained with a high degree of reliability (R^2^ = 0.992–0.998) for mass increments in water solution and in the salt solution (Figure 5, Table 5). There is little correlation between the deformation values in water and in solution (R^2^ = 0.133–0.980).

Table 5 shows the values of the slope coefficients and the R-squared value of the relationship between the mass increment of the samples and the values of their deformations in solution and in water at the same time points.

The presence of a correlation between the mass increment of the samples in water and in a Na_2_SO_4_ solution and the absence of such a relationship between changes in volume shows that these two processes are little related to each other. The mass increment is mainly determined by water consumption for cement hydration and absorption by the gel and contraction pores. The volume change is mainly due to contraction shrinkage, gel swelling, and the formation of expandable compounds. Direct proportionality here indicates that the Na_2_SO_4_ present in the solution, without practically affecting the hydration and associated processes, causes an additional water demand for the formation of hydrating compounds (gypsum and ettringite). In this case, the mass increment of the samples in the Na_2_SO_4_ solution in comparison with the mass increment in water increases with time in a constant percentage: for Mix No. 1 by 8%; for Mix No. 2 by 3%; for Mix No. 3 by 12%. In the case of the Control Mix, an inverse ratio of 10% is obtained. It is not possible to explain this circumstance within the framework of this article.

### 3.2. Strength Test Results

Another criterion for evaluating sulfate resistance, in addition to the expansion, is the strength of samples exposed to long-term aggressive environments.

After the end of the shrinkage–expansion experiments, after 192 days, the test beams with dimensions of 40 × 40 × 160 mm were left in the corresponding media for another 138 days, but at a higher temperature of 40 °C. Thus, some samples were hardened in water and other ones in 5% Na_2_SO_4_ solution for 6 months at a temperature of 20 °C and 4.6 months at a temperature of 40 °C.

Thereafter, the beams were tested for flexural strength at the age of 340 days. The halves of the beams were tested for compressive strength using standard 25 cm^2^ plates after the flexural strength test.

**Figure 6:**
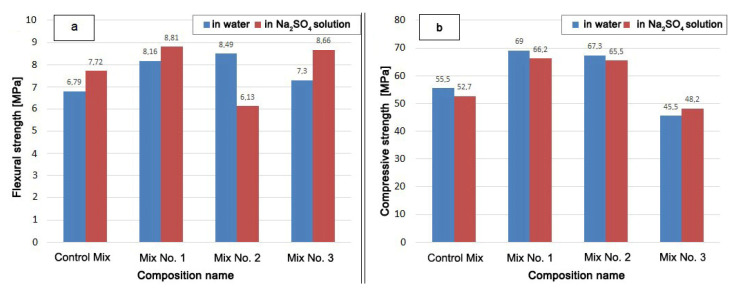
Sample strength after holding in water and in Na_2_SO_4_ solution for 340 days: (**a**) flexural strength; (**b)** compressive strength.

The strength test results are shown in Figure 6.

The mixture with the Zolest-bet additive (Mix No. 1) has the highest compressive strength after hardening both in water and in a Na_2_SO_4_ solution. The compressive strength of Mix No. 1 was 4% lower and the flexural strength 8% higher after hardened in Na_2_SO_4_ solution than after hardened in water. The strength of Mix No. 1 exceeded the compressive strength of the Control Mix by 24% (in water) and by 14% (in Na_2_SO_4_ solution).

The compressive strength of the mixture with silica fume additive (Mix No. 2) turned out to be slightly lower than that of the mixture with the Zolest-bet additive (Mix No. 1). This decrease was 2.5 and 1.0% in a water medium and in Na_2_SO_4_ solution, respectively. The flexural strength of Mix No. 2, hardened in solution, was significantly lower than when hardened in water. Mix No. 2 had the lowest value among the samples that had a positive effect on strength from the Na_2_SO_4_ solution. However, the compressive strength when exposed to Na_2_SO4 decreased for all mixtures, except for samples based on sulfate-resistant cement (Mix No. 3). The strength of Mix No. 3 in an aggressive environment increased both in the flexural strength and in the compressive strength.

### 3.3. Density Test Results

The density of the samples was calculated from the results of periodic weighing. Before the start of the experiment, the dimensions of the samples were determined with an accuracy of 0.1 mm. The change in the sample due to shrinkage or expansion was taken into account when determining the volume. The volume correction was calculated from the measured relative change in the sample length and the proportional change in the other two dimensions. However, this correction turned out to be negligible.

Table 6 shows the density values at the beginning of the experiment and after a 340-day immersion in water and in a 5% Na_2_SO_4_ solution.

The density of the samples before the experiment did not depend on the hardening environment, because in the first three days, the samples of both series were in the same conditions. As a result of staying in the appropriate environment for 340 days, the density of the Control Mix increased in comparison with the initial one by 1.3%. The density of the other mixtures increased in comparison with the initial one by 2.0–2.4%, regardless of the environment. The density after the experiment of the mixtures without additives (Control Mix and Mix No. 3), both in the water and in the Na_2_SO_4_ solution, turned out to be practically the same. The hardening conditions did not affect the density of mixtures with additives (Mix No. 1 and Mix No. 2). The density of these mixtures after being in the Na_2_SO_4_ solution turned out to be higher than the density after water hardening by 1.0 and 1.3%, respectively.

### 3.4. Assessment of the Appearance of Samples

**Figure 7:**
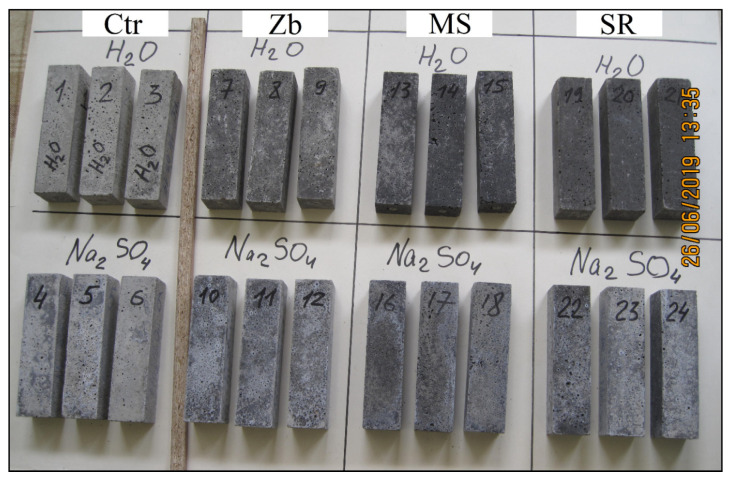
Photo of samples after 340 days in water and in Na_2_SO_4_ solution.

The samples hardened in Na_2_SO_4_ solution, in contrast to the samples in water, have a lighter uneven color due to white efflorescence (see Figure 7).

Structural damage in the form of cracks, delamination, and peeling of the surface were not found as a result of visual analysis of the samples.

## 4. Conclusions

1. The influence of the electrostatic precipitator ash “Zolest-bet” and silica fume on the sulfate resistance of Portland cement was studied. The evaluation criteria were the expansion, strength, density, and appearance of the samples, hardened in a 5% Na_2_SO_4_ solution starting from three days of age for 192 days at a temperature of 20 °C and 148 days at 40 °C. The test results were compared with the results of the mixture based on Portland cement without additives and a sulfate-resistant cement mixture.

2. A variable character of sample deformation was established, both in pure water and in a Na_2_SO_4_ solution. This consisted of a change in the periods of shrinkage and expansion, which may indicate competition between contraction shrinkage and expansion under the action of the formed compounds and the swelling gel. The degree of influence of Na_2_SO_4_ on expansion was estimated from the area between the deformation curves of the samples in a Na_2_SO_4_ solution and in pure water for 180 days. The mixture with the silica fume additive had the minimum expansion under the Na_2_SO_4_ action, and the mixture with the fly ash “Zolest-bet” additive had the greatest expansion according to this indicator.

3. A stable correlation between the expansion and the mass increment of the samples was not found in the experiments. A decrease in volume was observed when there was a mass increment. A directly proportional correlation between the values of mass increments in water and in Na_2_SO_4_ solution for the same mixtures at the same time points was established. There is no correlation between the values of the corresponding deformations in water and in solution.

4. Zolest-bet ash in pure water shrank the mixture by 0.19 mm/m by the end of the hardening period (180 days), and it gave a linear expansion of 0.23 mm/m (0.023%) in a Na_2_SO_4_ solution after hardening. The mixture can be considered sulfate resistant at a given value of linear expansion (norm ≤ 0.1%). All the more sulfate-resistant mixtures should be considered, as the remaining ones had less expansion than the fly ash mixture. Despite the greatest expansion, the compressive strength of the samples with the Zolest-bet additive was found to be the highest after hardening in both environments. The flexural strength was found to be the highest after being in Na_2_SO_4_ solution.

5. The sulfate resistance of the mixture with silica fume additive was higher than that of the mixture based on sulfate-resistant cement. This mixture did not expand in comparison with the initial length, but, on the contrary, it shrank, while the expansion of sulfate-resistant cement was 0.006% over the control period. The compressive strength of the mixture with the silica fume additive was slightly inferior to the strength of the mixture with Zolest-bet ash.

## Figures and Tables

**Table 1:** Mineralogical composition of the cement.

Cement	Mineralogical Composition (%)				Impurity Content (%)				Specific Surface Area (cm^2^/g)
	C_3_S	C_2_S	C_3_A	C_4_AF	MgO	SO_3_	Cl^−^	R_2_O	
CEM I 42.5 N	60.1	18.3	5.27	13.4	1.12	2.73	0.005	0.77	3420
CEM I 32.5 SR5	49.7	28.6	4.46	13.4	1.13	2.70	0.004	0.77	3440

**Table 2:** Chemical composition of the fly ash (%).

SiO_2_	CaO	CaO_free_	MgO	Fe_2_O_3_	Al_2_O_3_	SO_3_	K_2_O	Na_2_O	Loss on Ignition	Chloride
27.8	36.6	10.0	4.29	4.19	7.02	10.3	4.26	0.087	4.87	<0.1

**Table 3:** Concrete mixtures for sulfate durability test.

Composition Name	Type of Cement	Type of Additive	Material Content (g/L)				Cone Flow Diameter after 30 Shakes, mm
			Cement	Sand	Additive	Water	
Control Mix	CEM I 42.5 N	-	500	116	-	250	116
Mix No. 1	CEM I 42.5 N	“Zolest-bet”	400	135	100	250	135
Mix No. 2	CEM I 42.5 N	Silica fume	400	102	100	250	102
Mix No. 3	CEM I 32.5 N SR5	-	500	120	-	250	120

**Table 4:** Values of areas between deformation curves of samples in Na_2_SO_4_ solution and in water.

Mixture	Control Mix	Mix No. 1	Mix No. 2	Mix No. 3
**Area (d·mm/m)**	40.3	118.5	10.4	24.4

**Table 5:** Approximation parameters.

Composition Name	Correlation of Mass Increments		Correlation of Deformations	
	Slope Coefficient	R-Squared Value	Slope Coefficient	R-Squared Value
Control Mix	0.90	0.992	0.52	0.832
Mix No. 1	1.08	0.999	−0.51	0.554
Mix No. 2	1.03	0.995	0.69	0.980
Mix No. 3	1.12	0.998	0.47	0.133
All compositions	1.06	0.995	-	-

**Table 6:** Density test results.

Composition Name	Type of Cement	Type of Additive	Density (kg/m^3^)			
			Water Hardening		Na_2_SO_4_ Hardening	
			before Experiment	after Experiment	before Experiment	after Experiment
Control Mix	CEM I 42.5 N	-	2220	2249	2220	2248
Mix No. 1		“Zolest-bet”	2205	2254	2224	2276
Mix No. 2		Silica fume	2144	2189	2166	2217
Mix No. 3	CEM I 32.5 N SR5	-	2198	2242	2199	2247

## References

[1] Manalo A., Maranan G., Benmokrane B., Cousin P., Alajarmeh O., Ferdous W., Liang R., Hota G. (2020). Comparative durability of GFRP composite reinforcing bars in concrete and in simulated concrete environments. Cem. Concr. Compos..

[2] Majhi R.K., Nayak A.N., Mukharjee B.B. (2020). Characterization of lime activated recycled aggregate concrete with high-volume ground granulated blast furnace slag. Constr. Build. Mater..

[3] Zhao G., Shi M., Guo M., Fan H. (2020). Degradation mechanism of concrete subjected to external sulfate attack: Comparison of different curing conditions. Materials.

[4] ACI Committee 232 (2018). 232.2R-18: Report for the Use of Fly Ash in Concrete.

[5] Fediuk R.S., Mochalov A.V., Bituev A.V., Zayakhanov M.E. (2019). Structuring Behavior of Composite Materials Based on Cement, Limestone, and Acidic Ash. Inorg. Mater..

[6] Taskin A., Fediuk R., Grebenyuk I., Elkin O., Kholodov A. (2020). Effective cement binders on fly and slag waste from heat power industry of the primorsky krai, Russian federation. Int. J. Sci. Technol. Res..

[7] Barabanshchikov Y., Fedorenko I., Kostyrya S., Usanova K. (2019). Cold-Bonded Fly Ash Lightweight Aggregate Concretes with Low Thermal Transmittance: Review. Adv. Intell. Syst. Comput..

[8] Usanova K., Barabanshchikov Y.G. (2020). Cold-bonded fly ash aggregate concrete. Mag. Civ. Eng..

[9] Plowman C., Cabrera J.G. (1996). The use of fly ash to improve the sulphate resistance of concrete. Waste Manag..

[10] Bhatt A., Priyadarshini S., Acharath Mohanakrishnan A., Abri A., Sattler M., Techapaphawit S. (2019). Physical, chemical, and geotechnical properties of coal fly ash: A global review. Case Stud. Constr. Mater..

[11] Fediuk R., Smoliakov A., Cherkasov A., Bezruk G., Evseev A. (2020). Application of Household Waste as Aggregates for Concrete. IOP Conf. Ser. Mater. Sci. Eng..

[12] Bazhenov Y.M., Zagorodnjuk L.H., Lesovik V.S., Yerofeyeva I.V., Chernysheva N.V., Sumskoy D.A. (2016). Concerning the role of mineral additives in composite binder content. Int. J. Pharm. Technol..

[13] Rybakov V., Seliverstov A., Petrov D., Smirnov A., Volkova A. (2018). Strength characteristics of foam concrete samples with various additives. MATEC Web Conf..

[14] Rybakov V., Seliverstov A., Petrov D., Smirnov A., Volkova A. (2018). Lightweight steel concrete structures slab panels load-bearing capacity. MATEC Web Conf..

[15] Shashiprakash S.G., Thomas M.D.A. (2001). Sulfate Resistance of Mortars Containing High-Calcium Fly Ashes and Combinations of Highly Reactive Pozzolans and Fly Ash. Symp. Pap..

[16] Schlorholtz S., Bergeson K., Demirel T. (1987). Monitoring of Fluctuations in the Physical and Chemical Properties of a High-Calcium Fly Ash. MRS Proc..

[17] Kang S., Tyler Ley M., Lloyd Z., Kim T. (2020). Using the Particle Model to predict electrical resistivity performance of fly ash in concrete. Constr. Build. Mater..

[18] Soleymani Ashtiani M., Scott A.N., Dhakal R.P. (2013). Mechanical and fresh properties of high-strength self-compacting concrete containing class C fly ash. Constr. Build. Mater..

[19] Raado L.M., Hain T., Liisma E., Kuusik R. (2014). Composition and properties of oil shale ash concrete. Oil Shale.

[20] Gulbe L., Setina J., Juhnevica I. (2017). The use of shale ash in dry mix construction materials. IOP Conf. Ser. Mater. Sci. Eng..

[21] Mahadin S., Al-Hamaiedeh H., Maaitah O. (2010). Using Oil Shale Ash in Concrete Binder. Electron. J. Geotech. Eng..

[22] Reid D., Vimmr V. Concrete mix utilising oil shale ash. Proceedings of the Concrete Structures for Sustainable Community—Proceedings, Fib Symposium 2012.

[23] Kaewmanee K., Krammart P., Sumranwanich T., Choktaweekarn P., Tangtermsirikul S. (2013). Effect of free lime content on properties of cement–fly ash mixtures. Constr. Build. Mater..

[24] Hemalatha T., Ramaswamy A. (2017). A review on fly ash characteristics—Towards promoting high volume utilization in developing sustainable concrete. J. Clean. Prod..

[25] Klyuev S.V., Klyuev A.V., Khezhev T.A., Pukharenko Y.V. (2018). Technogenic sands as effective filler for fine-grained fibre concrete. J. Phys. Conf. Ser..

[26] Gooi S., Mousa A.A., Kong D. (2020). A critical review and gap analysis on the use of coal bottom ash as a substitute constituent in concrete. J. Clean. Prod..

[27] Gopalan M.K. (1996). Sorptivity of fly ash concretes. Cem. Concr. Res..

[28] Xie J., Kayali O. (2016). Effect of superplasticiser on workability enhancement of Class F and Class C fly ash-based geopolymers. Const. Build. Mater..

[29] Zibarev P.V., Zubkova O.A., Shepelenko T.S., Nedavnii O.I. (2006). Gas chromatographic control of toxic organic microimpurities in water via the method of concentration on modified porous polymer sorbents. Russ. J. Nondestruct. Test..

[30] Abousnina R., Manalo A., Ferdous W., Lokuge W., Benabed B., Saif Al-Jabri K. (2020). Characteristics, strength development and microstructure of cement mortar containing oil-contaminated sand. Constr. Build. Mater..

